# The complete mitochondrial genome of the stomatopod crustacean *Squilla mantis*

**DOI:** 10.1186/1471-2164-6-105

**Published:** 2005-08-09

**Authors:** Charles E Cook

**Affiliations:** 1 Department and Museum of Zoology, University of Cambridge, Downing Street, Cambridge, CB2 3EJ, UK; 2 Natural Environment Research Council, British Antarctic Survey, Biological Sciences Division, High Cross, Madingley Road, Cambridge CB3 0ET, UK

## Abstract

**Background:**

Animal mitochondrial genomes are physically separate from the much larger nuclear genomes and have proven useful both for phylogenetic studies and for understanding genome evolution. Within the phylum Arthropoda the subphylum Crustacea includes over 50,000 named species with immense variation in body plans and habitats, yet only 23 complete mitochondrial genomes are available from this subphylum.

**Results:**

I describe here the complete mitochondrial genome of the crustacean *Squilla mantis *(Crustacea: Malacostraca: Stomatopoda). This 15994-nucleotide genome, the first described from a hoplocarid, contains the standard complement of 13 protein-coding genes, 22 transfer RNA genes, two ribosomal RNA genes, and a non-coding AT-rich region that is found in most other metazoans. The gene order is identical to that considered ancestral for hexapods and crustaceans. The 70% AT base composition is within the range described for other arthropods. A single unusual feature of the genome is a 230 nucleotide non-coding region between a serine transfer RNA and the *nad1 *gene, which has no apparent function.

I also compare gene order, nucleotide composition, and codon usage of the *S. mantis *genome and eight other malacostracan crustaceans. A translocation of the histidine transfer RNA gene is shared by three taxa in the order Decapoda, infraorder Brachyura; *Callinectes sapidus, Portunus trituberculatus *and *Pseudocarcinus gigas*. This translocation may be diagnostic for the Brachyura. For all nine taxa nucleotide composition is biased towards AT-richness, as expected for arthropods, and is within the range reported for other arthropods. Codon usage is biased, and much of this bias is probably due to the skew in nucleotide composition towards AT-richness.

**Conclusion:**

The mitochondrial genome of *Squilla mantis *contains one unusual feature, a 230 base pair non-coding region has so far not been described in any other malacostracan. Comparisons with other Malacostraca show that all nine genomes, like most other mitochondrial genomes, share a bias toward AT-richness and a related bias in codon usage. The nine malacostracans included in this analysis are not representative of the diversity of the class Malacostraca, and additional malacostracan sequences would surely reveal other unusual genomic features that could be useful in understanding mitochondrial evolution in this taxon.

## Background

The mitochondria are extranuclear organelles present in all metazoans. They contain a circular genome, usually around 16 kilobases in length, with 37 genes (13 protein-coding, two ribosomal RNA genes, and 22 transfer RNA genes). This gene content is widely conserved, but gene order and the DNA sequences of the genes themselves are variable. Because of their small size many more mitochondrial genomes than nuclear genomes have been sequenced, and comparisons among them may serve as models for the evolution of the much larger nuclear genomes [1]. In addition, gene order rearrangements and mitochondrial gene sequences have been widely used for phylogenetic inference [2-7].

At present there are about 650 complete mitochondrial genomes available in public databases. Of these, about 75 percent are of vertebrates. By contrast only 129 complete mitochondrial genomes are available from arthropods, which are the most diverse and speciose phylum of animals. In addition, there is considerable taxonomic bias among the available arthropod sequences; 86, (67 percent) are hexapods. The subphylum Crustacea includes over 50,000 named species and is ecologically and morphologically the most diverse of the arthropod groups, and therefore of all the animals. Crustaceans occupy marine, terrestrial, and fresh water habitats from the deep sea to high mountains; range in adult size from less than one millimeter to more than four meters (leg span); and exhibit extensive variability in body plans when compared to other arthropod groups [8]. At present there are 23 complete crustacean mitochondrial genomes available. Within the Crustacea members of the class Malacostraca, which include crabs, lobsters, and shrimp, are perhaps the most well known to non-scientists. Due to their economic importance this group is often the focus of scientific enquiry. At present there are nine complete malacostracan mitochondrial genomes available, including that of the stomatopod shrimp *Squilla mantis*. In this paper I describe this genome and compare it to eight other mitochondrial genomes that are available from other Malacostraca.

Mantid shrimps, or stomatopods, are benthic predators distributed in the shallow waters of tropical and subtropical seas. They are best known for their raptorial appendages – pointed or clubbed – which they use to make lightning-fast attacks that disable prey animals by spearing or blunt trauma. Large individuals with the club-type appendages have been known to shatter the sides of aquaria [9]. *Squilla mantis *(Linnaeus, 1758) (Crustacea: Malacostraca: Stomatopoda), with a maximum length of around 20 cm, is distributed in shallow waters throughout the Mediterranean Sea and Eastern Atlantic [10]. *S. mantis *is widely consumed by humans throughout its range; UN Food and Agriculture Administration statistics indicate that total catches in the Mediterranean are currently in excess of 6500 tonnes per annum so this species is of some commercial importance [11].

## Results and discussion

### Mitochondrial genome composition

The mitochondrial genome of *Squilla mantis *(GenBank accession number AY639936) is a circular molecule of 15994 nucleotides that contains the same 13 protein-coding genes, 22 transfer RNA genes (tRNA), and two ribosomal RNA genes (rRNA) found in other metazoans [12,13]; the majority strand (i.e., the strand encoding the majority of genes) encodes nine protein-coding genes and 14 tRNAs while the minority strand encodes four protein-coding genes, eight tRNAs and both rRNA genes (Table 1). The *S. mantis *genome, like that reported for other arthropod genomes, is AT-rich and has an overall AT content of 70%. This frequency, as expected, varies for different regions of the genome. First and second codon positions average 62% and 63% AT, respectively, tRNA and rRNA genes average 72%, third codon positions average 79%, and putative non-coding regions reach up to 87% AT content (Table 2). There are no significant differences in AT frequency for genes encoded on the majority or minority strands. These values are within the range of 60–87% reported for other arthropods and are not unusual [14,15].

**Table 1:** Complete mitochondrial genomes available for the Malacostraca. Taxononomic classification from the NCBI taxonomy browser [37] and from reference [8].

Species	**GenBank accession**	**Order**	**Infraorder/Suborder**	**Family**
*Callinectes sapidus*	NC_006281	Decapoda	Brachyura	Portunidae
*Cherax destructor*	NC_011243	Decapoda	Astacidea	Parastacidae
*Harpiosquilla harpax*	AY699271	Stomatopoda	Unipeltata	Squillidae
*Macrobrachium rosenbergii*	AY659990	Decapoda	Caridea	Palaemonidae
*Pagurus longicarpus*	NC_003058	Decapoda	Anomura	Paguridae
*Panulirus japonicus*	NC_004251	Decapoda	Palinura	Palinuridae
*Penaeus monodon*	NC_002184	Decapoda	Dendrobranchiata	Penaeidae
*Portunus trituberculatus*	NC_005037	Decapoda	Brachyura	Portunidae
*Pseudocarcinus gigas*	AY562127	Decapoda	Brachyura	Eriphiidae
*Squilla mantis*	NC_006081	Stomatopoda	Unipeltata	Squillidae

**Table 2:** Nucleotide composition of Squilla mantis mitochondrial genome features. Major and minor strand genes show no significant differences in nucleotide composition.

**Genome feature**	**Number of nucleotides**	**Proportion of nucleotides**				**%AT**
		**A**	**C**	**G**	**T**	
All nucleotides (major strand)	15994	0.351	0.168	0.130	0.351	70
rRNA genes (minor strand)	1760	0.464	0.193	0.135	0.208	67
AT-rich region 1 (major strand)	230	0.448	0.061	0.065	0.426	87
AT-rich region 12(major strand)	861	0.423	0.137	0.095	0.345	77
tRNA genes (major strand)	945	0.361	0.126	0.157	0.357	72
tRNA genes (minor strand)	469	0.360	0.104	0.177	0.358	72
tRNA genes (both strands)	1414	0.361	0.119	0.163	0.357	72
Unassigned nucleotides (major strand)	76	0.316	0.132	0.013	0.539	86
***Protein coding genes***						
All positions	11070	0.274	0.156	0.174	0.396	67
first codon positions	3690	0.292	0.136	0.241	0.331	62
second codon positions	3690	0.187	0.208	0.162	0.444	63
third codon positions	3690	0.359	0.106	0.102	0.433	79
*Majority strand protein coding genes*						
All positions	6966	0.278	0.182	0.163	0.377	66
first codon positions	2262	0.287	0.154	0.242	0.317	60
second codon positions	2262	0.190	0.237	0.140	0.434	62
third codon positions	2262	0.380	0.130	0.079	0.411	79
*Minority strand protein coding genes*						
All positions	4284	0.269	0.113	0.191	0.427	70
first codon positions	1428	0.300	0.107	0.239	0.353	65
second codon positions	1428	0.182	0.162	0.195	0.460	64
third codon positions	1428	0.325	0.068	0.138	0.469	79

### Gene structure

**Figure 1:**
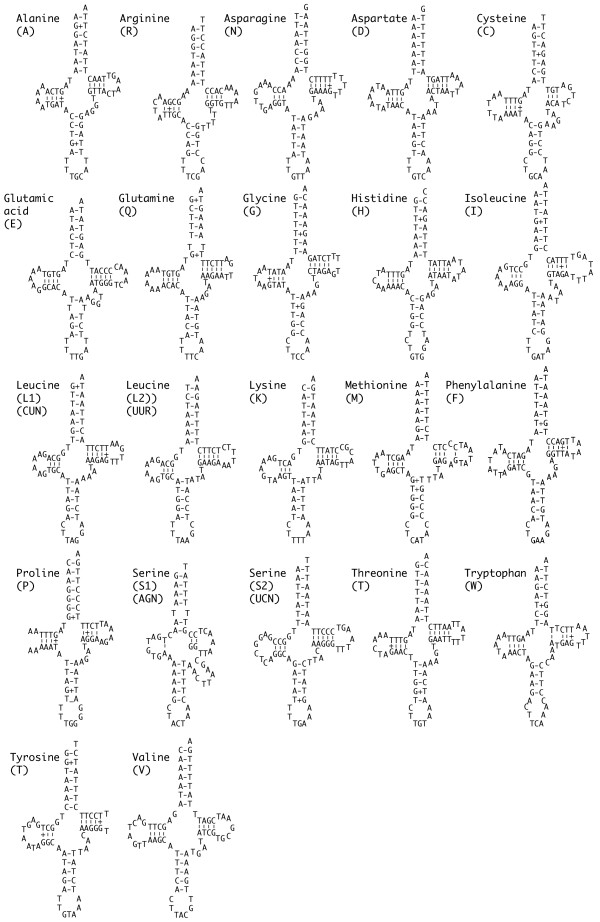
*Squilla mantis *mitochondrial tRNA genes folded into inferred cloverleaf structures.

The predicted structures of the 22 *S. mantis *tRNAs are shown in Figure 1. Twenty one of these genes were identified by tRNAscan-SE [16] and have secondary structures similar to those of other published metazoan tRNA genes. Two genes, *trnS*_1 _and *trnQ*, have single T-T mismatches in the acceptor stem, and one gene, *trnM*, has a single C-A mismatch in the stem of the TψC loop. The *trnS*_1 _gene was not identified by the tRNAscan software; rather, it was located by its conserved position in the genome. The variable loop of this gene, with nine nucleotides, is longer than the average of four or five for mitochondrial tRNA genes. This feature is characteristic of type 2 transfer RNA genes, which are uncommon in animal mitochondria but are the norm for bacterial and eukaryotic *trnS *genes.

The large and small subunit ribosomal RNA (rRNA) genes (*rnl*, *rns*) have an AT content of 67%, within the range reported for other arthropod ribosomal RNA genes. Alignments of these genes with other arthropod homologues (not shown) as expected show both conserved and unconserved regions that correspond with the putative stems and loops within these genes. There are thus no unusual features to report for the two rRNA genes.

All of the 13 protein-coding genes, except *cox1*, have putative ATR methionine or ATT isoleucine start codons. The putative first codon of the *cox1 *gene is ACG threonine. The lack of a standard initiation codon in *cox1 *genes is common in arthropod mitochondria, so *S. mantis *is not unusual [17,18]. Two of the protein-coding genes, *cox1 *and *nad6*, lack a full TAA or TAG stop codon. These genes appear to terminate with a single T from which a stop codon is created by polyadenylation of the mRNA during processing. Again, this phenomenon has been observed in other arthropod mitochondrial genomes and is not unusual [17,19,20].

### AT-rich regions

Arthropod mitochondrial genomes typically have a long region that has an AT content higher than that of mitochondrial coding regions. This AT-rich region, ranging from 263 to 4601 base pairs in length and usually located between the *rns *and *trnI *genes, is often termed the control region because it contains a number of regulatory elements including the origin of replication for the heavy strand of the mitochondrial genome [21,22]. In some arthropods the AT-rich region is reported to have any or all of these four different motifs: tandemly repeated sequences, a long sequence of T's, a subregion of even higher AT richness, and stem-loop structures [23,24].

In *S. mantis *there are two AT-rich regions, numbered 1 and 2 on Tables 2 and 3. AT-rich region 2 corresponds to the conventional arthropod region between *rns *and *trnI*; it is 862 base pairs long, well within the reported range for other arthropods, with an AT content of 77% compared to 70% for the entire *S. mantis *mitochondrion. However, this region has none of the four motif types that have been reported for arthropods, and I was not able to identify any putatively functional motifs.

**Table 3:** Annotation of the mitochondrial genome of Squilla mantis. Position numbers refer to positions on the major strand, with the first nucleotide of *trnI *assigned as number 1. Parentheses indicate genes encoded on the minor strand. Intergenic region numbers refer to the number of non-coding bases between features (positive integers) or the number of overlapping bases (negative integers). Asterisks (*) refer to incomplete termination codons that may be extended by post-transcriptional adenylation.

**Feature**	Position	**First codon**	**Stop codon**	**Intergenic region**
*trnI*	1–69			
*trnQ*	(68–135)			-2
*trnM*	149–217			13
*nad2*	275–1219	ATA	TAA	57
*trnW*	1218–1285			-2
*trnC*	(1291–1354)			5
*trnY*	(1356–1420)			1
*cox1*	1421–2959	ACG	TAA	0
*trnL2*	2955–3021			-5
*cox2*	3026–3713	ATG	T*	0
*trnK*	3714–3781			0
*trnD*	3781–3849			-1
*atp8*	3850–4002	ATC	TAA	0
*atp6*	3996–4673	ATG	TAA	-7
*cox3*	4673–5461	ATG	TAG	-1
*trnG*	5461–5526			-1
*nad3*	5527–5880	ATG	TAA	0
*trnA*	5884–5948			3
*trnR*	5953–6017			4
*trnN*	6019–6087			1
*trnS1*	6088–6155			0
*trnE*	6156–6221			0
*trnF*	(6222–6288)			0
*nad5*	(6289–8001)	ATA	TAA	0
*trnH*	(8020–8086)			18
*nad4*	(8086–9426)	ATG	TAG	-1
*nad4L*	(9420–9719)	ATG	TAA	-7
*trnT*	9722–9790			2
*trnP*	(9791–9857)			0
*nad6*	9900–10386	ATA	T*	42
*cob*	10387–11523	ATG	TAA	0
*TrnS2*	11523–11592			-1
AT-rich region 1	11593–11822			0
*nad1*	(11823–12803)	ATA	TAA	0
*trnL1*	(12801–12867)			-3
*rnl*	(12868–14250)			0
*trnV*	(14251–14300)			0
*rns*	(14301–15132)			0
AT-rich region 2	15133–15994			0

I therefore examined the shorter AT-rich region of 230 nucleotides between the *trnS*_2 _and *nad1 *genes for possible functional motifs. Most arthropod mitochondrial genomes have a few short non-coding regions between some genes, usually from a few bases to 20 bases long, but longer non-coding regions, such as AT-rich region 1 in *S. mantis*, are rare. It therefore seemed possible that this region might have taken over some of the functions putatively assigned to the longer AT-rich region. However, AT-rich region 1, like region 2, contains none of the motifs listed above. Furthermore, the AT content of this region, at 87%, is similar to that calculated collectively for other unassigned nucleotides in the *S. mantis *genome (Table 2) and is consistent with the hypothesis that this region has no function.

Unusual genomic features, such as this non-coding region or gene order rearrangements, can be useful as characters for reconstructing evolutionary relationships [13,25]. A second AT-rich region is notably absent even in *Harpiosquilla harpax*, which is also a member of the family Squillidae. A survey of other members of the genus *Squilla *for the presence of a similar region would perhaps enhance our understanding of the history of this unusual genomic feature.

### Comparison with other malacostracan crustaceans

A number of features of mitochondrial genomes can be used to infer relationships among taxa. These include phylogenetic analysis using DNA and protein sequences, relative rates of sequence evolution, gene order, and the effective number of codons (N_c_). I present a phylogenetic analysis and a discussion of rates of sequence evolution in arthropods (including *S. mantis*) elsewhere [26], and discuss gene order and N_c _below.

**Figure 2:**
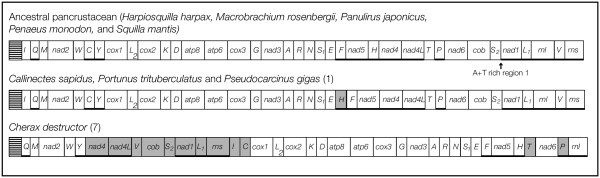
Mitochondrial gene orders for nine malacostracan crustaceans. The style of the figure is adapted from Figure 1 of Lavrov et al. [25]. Protein and ribosomal RNA genes (large boxes) are abbreviated as in the text. Transfer RNA genes are abbreviated with single letter codes (see Figure 1). The striped box represents the AT-rich region. The ancestral pancrustacean gene order is found for five of the nine taxa, including *Squilla mantis*. The position of AT-rich region 1 in the *S. mantis *genome is noted with an arrow. Genes are transcribed from right to left except when underlined. Shaded boxes indicate genes whose positions differ from their positions in the ancestral pancrustacean sequence. The number in parentheses next to taxa names represents the minimum number of rearrangement events that separates that gene arrangement from the ancestral pancrustacean gene order (see Miller et al. [20] for a fuller discussion of the rearrangements in *C. destructor*).

Rearrangements of the mitochondrial genome are relatively rare events in evolutionary history. Such rare events can be used to infer relationships among taxa, and mitochondrial gene order rearrangements have proven useful in understanding some aspects of arthropod evolution [27-29]. Figure 2 shows the mitochondrial gene order for the nine Malacostraca for which there are complete mitochondrial genomes. Five of these genomes share the gene order considered ancestral for the Pancrustacea (Crustacea + Hexapoda) [27]. *Callinectes sapidus, Portunus trituberculatus *and *Pseudocarcinus gigas *share a single translocation of *trnH *compared to the ancestral gene order. The mitochondrial genome of *Cherax destructor *is considerably rearranged and evidences at least seven translocation events compared to the ancestral pancrustacean arrangement [20]. *C. sapidus, P. trituberculatus *and *P. gigas *are all decapods within the infraorder Brachyura (Table 1). The *trnH *translocation shared by these three taxa is therefore not surprising. It is possible that this character is shared among all of the Brachyura, and could therefore serve as a marker for membership in this group and might aid in rapid identification of unidentified individuals, such as larvae or processed materials in markets.

The effective number of codons used in a gene, N_c_, is a statistic developed by Wright [30] to quantify how far codon usage in a gene departs from the equal use of all synonymous codons. The value of N_c _can range from 20, the theoretical extreme in which only one codon is used for each amino acid, to 61 when the use of all synonymous codons is equally likely. This statistic, initially developed to compare codon usage between different genes in the same genome, can also be used to compare codon usage between genomes. I calculated N_c _for each of the nine malacostracan mitochondrial genomes using the program CodonW [31]. Table 4 shows N_c _and GC content for majority strand, minority strand, and all protein genes in the malacostracan mitochondrial genomes. GC rather than AT content is presented to conform to the convention for these comparisons. The N_c _values, which range from 38 to 53, are all below the value of 61 that indicates random codon usage, so codon usage in all nine genomes is non-random. There are no obvious similarities in the values for related taxa (i.e., the decapods), but extensive additional sampling among the Malacostraca would be necessary to confirm this observation.

**Table 4:** Codon usage in nine malacostracan mitochondrial genomes. Columns indicate GC content and effective number of codons (N_c_) for majority strand genes, minority strand genes, and all genes. The least squares linear regression equation and the coefficient of determination are also shown for each data set.

**Taxon**	**Majority strand genes**		**Minority strand genes**		**All genes**	
	**N**_c_	**GC**	**N**_c_	**GC**	**N**_c_	**GC**

*Callinectes sapidus*	43.36	0.348	39.05	0.302	44.79	0.33
*Cherax destructor*	52.79	0.423	49.65	0.366	55.25	0.401
*Harpiosquilla harpax*	40.17	0.325	40.23	0.309	41.08	0.319
*Macrobrachium rosenbergii*	43.92	0.418	39.74	0.369	52.49	0.399
*Pagurus longicarpus*	38.17	0.319	34.85	0.279	39.6	0.304
*Panulirus japonicus*	49.71	0.382	49.8	0.361	51.3	0.374
*Penaeus monodon*	39.79	0.322	34.99	0.284	39.44	0.307
*Portunus trituberculatus*	40.71	0.326	37.29	0.289	40.97	0.312
*Pseudocarcinus gigas*	48.57	0.332	49.89	0.308	45.96	0.315
*Squilla mantis*	42.48	0.328	38.45	0.303	41.57	0.319
Least sqaures equation	y = 84.83x+14.08		y = 116.49x+4.47		y = 145.8x-4.044	
Coefficient of determination (R^2^)	R^2 ^= 0.5015		R^2 ^= 0.4493		R^2 ^= 0.9139	

**Figure 3:**
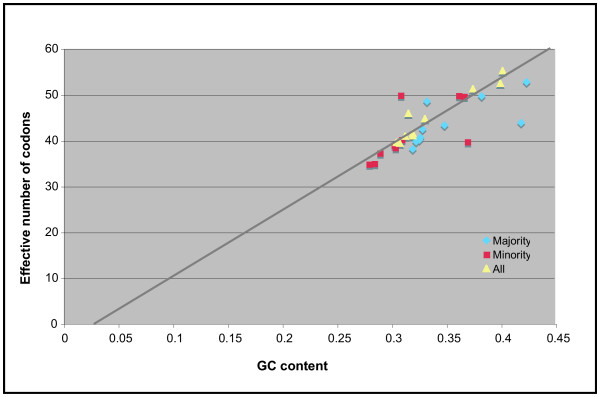
Relationship between GC content and effective number of codons for mitochondrial protein-coding genes. The line represents the least squares linear regression calculated for the all genes data set. This equation is shown in Table 4.

In Figure 3 N_c _and GC values are plotted. The distribution of the points suggests a linear relationship between N_c _and GC content. A similar association of N_c _and GC content was observed by Negrisolo *et al*. [32]. Equations representing a least squares linear regression analysis are shown for all three data sets in Table 2. Only the regression line for the all genes data set is shown on Figure 3 to prevent clutter. These equations are not statistically probative, but the distribution of points around the line shown in Figure 3 does add to the qualitative impression of a relationship between N_c _and GC content. I also calculated the coefficient of determination (R^2^) for each data set. The values for the majority and minority strand columns are near 0.5, suggesting that around 50% of the variation in one variable is associated with the other. That is, if GC content is taken as independent then 50% of the codon bias in the majority and minority strands is due to the influence of the bias towards low GC values. When both data sets are combined R^2 ^rises to 0.91, suggesting that codon bias and GC content are very closely associated. This discordance between R^2 ^for each strand separately and R^2 ^for all protein-coding genes is puzzling and merits additional study. However, if one assumes that GC content is driven by other biochemical factors then it is clear that much, if not most, of the codon bias observed in these mitochondrial genomes is a consequence of this nucleotide bias.

## Conclusion

This is the first formal description of the mitochondrial genome of a stomatopod crustacean. This genome maintains the same genes and gene order that are inferred as ancestral in the Pancrustacea, but does contain one unusual feature: a 230 base pair AT-rich region between the *trnS*_2 _and *nad1 *genes. This feature has no discernable function, but it may prove useful as a character in understanding the evolutionary history of the genus *Squilla*. Three other arthropod mitochondrial genomes have two large non-coding regions; the ostracod crustacean *Vargula hilgendorfii*, a millipede *Thyropygus *sp., and the tick *Boophilus microplus *[15,33,34]. Of these the latter two are clearly duplications of the orginal control region. Only *V. hilgendorfii *has, like *S. mantis*, two apparently unrelated non-contiguous AT-rich regions.

A comparison of nine malacostracan genomes, including that of *S. mantis*, shows that all nine exhibit the nucleotide composition bias favoring A and T nucleotides that is commonly observed for arthropod genomes, and that this bias is responsible at least in part for the observed codon usage bias in these genomes. However, there are no observable patterns of nucleotide composition bias or codon usage bias that unite particular taxa into common groups. These nine taxa represent only a small fraction of the diversity of the Malacostraca, and additional sequencing from across the diversity of this taxon would provide additional data for understanding the evolution of mitochondrial genomes of the class.

## Methods

### Samples and DNA extraction

A single freshly caught specimen of *S. mantis *was purchased from the fish market at Heraklion, Crete, Greece. Six grams of abdominal muscle were dissected from the specimen and immediately frozen at -70°C. Approximately one half gram of the frozen tissue was shaved from the specimen using a sterile razor blade and genomic DNA extracted using a QIAGEN genomic-tip 20 and the associated buffer set according to the manufacturer's protocol.

### PCR, sequencing, and annotation

Short fragments (300–1000 nucleotides) of the mitochondrial genome were amplified at low stringency (50–55 degree annealing temperatures) using primers designed to work on all arthropods (Table 4). Amplification products were cloned into the T-Easy vector (Promega) and at least three clones from each PCR product were sequenced with vector-specific primers using ABI Big-Dye chemistry. *Squilla*-specific primers were designed and used to amplify longer fragments of 1000–4000 nucleotides that spanned the gaps between the short fragments. Longer fragments were also ligated into the T-Easy vector and at least three clones from each ligation were isolated. Each clone was sequenced using a primer-walking strategy initiated with vector-specific primers. Sequences were assembled using Sequencher v. 3.1 (GeneCodes Corp.). Protein-coding genes were identified using BLAST searches [35] and by comparison with other arthropod mitochondrial genome sequences. Transfer RNA genes were identified using tRNAscan-SE [36]. Transfer RNA sequences were folded by eye, but made use of the tRNAscan-SE server output when that was available. The effective number of codons, N_c _was calculated using the software package CodonW [31].

## Abbreviations

Protein genes: cox1, cox 2, cox 3, cytochrome oxidase subunits I, II, and III; cob, cytochrome oxidase b; atp6, atp8, ATP synthase subunits 6 and 8; nad1, nad2, nad3, nad4, nad4L, nad5, nadD6, NADH dehydrogenase subunits 1–6 and 4L. Large and small subunit ribosomal RNA genes are abbreviated rnl and rns. Transfer RNA genes are listed as trnA, trnC, etc., where the final letter is the single letter abbreviation for that amino acid. This nomenclature follows that of Lavrov et al. [25].

**Table 5:** Primers used to amplify short fragments of the *Squilla mantis *genome. Majority/minority strand are with reference to the ancestral pancrustacean sequence. These primers will work for almost all arthropods and for many other metazoans as well. Reference "JB" is J. Boore pers. comm. Reference "CEC" refers to primers designed for this project.

**Gene**	**Majority strand**	**Sequence**	**Minority strand**	**Sequence**	**Reference**
*COX1*	Lco1490	GGTCAACAAATCATAAAGATATTGG	Hco2198	TAAACTTCAGGGTGACCAAAAAATCA	38
*COX1-COX2*	CO1DL	CCWCGWCGWTAYTCWGAYTAYCCWGA	CO2DL	CWGAATARRCATAWSWTCARTATCATTG	JB
*ATP6-COX3*	ATP6.AANMM	GCCGTACGGCTTGCAGCNAAYATRAT	CO3DL2	ACWACGTCKACGAAGTGTCARTATCA	CEC, JB
*COX3*	CO3DL1	TGGTGGCGAGATGTKKTNCGNGA	CO3DL2	ACWACGTCKACGAAGTGTCARTATCA	JB
*ND5*	ND5-R-DL	TARAAKCCWGMTARAAAWGGKAWWCC	ND5-F-DL	TWYTATTAGGKTGAGATGGKYTNGG	JB
*ND4*	ND4-R_DL	GARGAWCAKAWWCCRTGAGCAATYAT	ND4-F-DL	CCKAARGCYCAYGTKGARGCYCC	JB
*CYTB*	Cytb424-449	GGWTAYGTWYTWCCWTGRGGWCARAT	Cytb876-847	GCRTAWGCRAAWARRAARTAYCAYTCWGG	JB
*ND1*	ND1DL1	CCTTCWGCAAAATCGAAAGGGGYHCG	ND1.YIQIR	AAGATCCTTGGATAYATYCARATYCG	JB, CEC
*ssrRNA*	12Sb-3'	GAGGGTGACGGGCGGTGTGT	12sa-5'	AAACTGGGATTAGATACCCTATTAT	JB
*lsrRNA*	16SBRH	CCGGTCTGAACTCAGATCACGT	16SARL	CGCCTGTTTATCAAAAACAT	39
